# Supplementary material for: Shifts in stability and control effectiveness during evolution of Paraves support aerial maneuvering hypotheses for flight origins
Source: PeerJ. 2014 Oct 16;2:e632. doi: 10.7717/peerj.632 (PMC4203027; doi:10.7717/peerj.632)
Supplement: Supplemental Information 1 [file peerj-02-632-s001.pdf]

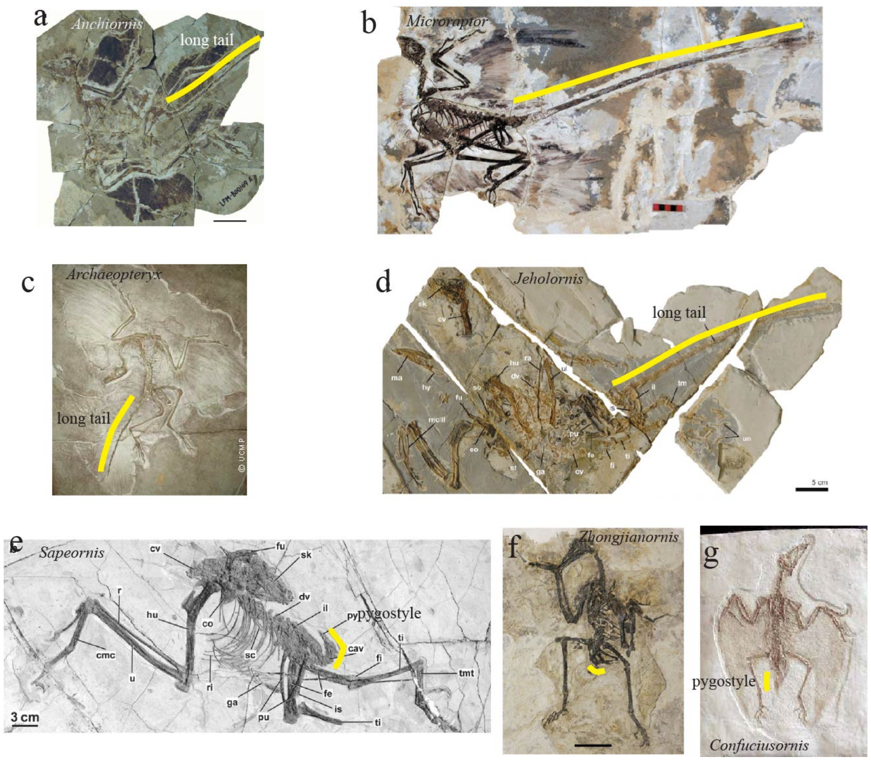

Figure S1. Fossils paravians (Xu et al., 2011) used for comparative study. (a) *Anchiornis* (Hu et al., 2009); (b) *Microraptor* (Xu et al., 2003; Li et al., 2012); (c) *Archaeopteryx* (Benton, 2005; Longrich, 2006), (d) *Jeholornis* (Zhou et al., 2002; Zhou and Zhang, 2003b; O'Connor et al., 2012; O'Connor et al., 2013), (e) *Sapeornis* (Zhou and Zhang, 2003b; Zheng et al., 2013), (f) *Zhongjianornis* (Zhou and Li, 2010) and (g) *Confuciusornis* (Gao et al., 2008; Hou et al., 1995; Chiappe et al., 1999). Subsequent specimens suggest *Sapeornis* has a long tail and a more basal phylogenetic placement than *Jeholornis* (Zheng et al., 2013; Turner et al., 2012).

Table S1. (a) Pitch static stability and equilibrium point, (b) control effectiveness in pitch using tail dorsiflexion (Figure 3b), (c) control effectiveness in pitch using symmetric wing protraction/retraction (wing sweep, Figure 3a). Results give mean ± s.d., $n = 15$ for stability, $n = 5$ for control effectiveness; units for stability and control effectiveness are rad^{-1}.

	a				
	pitch stability, $dC_m/d\alpha$			equilibrium point	
	$\alpha = 0°$	$\alpha = 15°$	$\alpha = 75°$	$\alpha°$	equilibrium $dC_m/d\alpha$
Anchiornis	-0.005 ±0.013	-0.067 ±0.012	-0.13 ±0.03	29 ± 2	-0.170 ±0.028
Archaeopteryx	-0.134 ±0.013	-0.221 ±0.020	-0.18 ±0.03	9 ± 2	-0.190 ±0.012
Confuciusornis	0.142 ±0.009	0.039 ±0.020	-0.06 ±0.02	0 ±2	0.142 ±0.009
Jeholornis	0.011 ±0.018	-0.108 ±0.010	-0.10 ±0.02	25 ±1	-0.120 ±0.044
Microraptor	-0.039 ±0.006	-0.071 ±0.009	-0.17 ±0.02	20 ± 2	-0.070 ±0.029
Sapeornis	0.109 ±0.010	0.007 ±0.011	-0.11 ±0.02	5 ± 2	0.100 ±0.052
Zhongjianornis	0.305 ±0.036	0.195 ±0.027	-0.15 ±0.16	5 ±1	0.180 ±0.078
Alectoris	0.206 ±0.006	0.090 ±0.020	-0.37 ±0.16	0 ± 1	0.120 ±0.013
Buteo	0.187 ±0.010	-0.042 ±0.009	-0.13 ±0.02	0 ± 3	0.140 ±0.030
Columba	0.046 ±0.014	-0.047 ±0.017	-0.18 ±0.16	0 ± 6	0.050 ±0.088
Larus	0.352 ±0.028	0.092 ±0.015	-0.10 ±0.02	0 ± 1	0.150 ±0.049
Onychonycteris	-0.011 ±0.011	0.112 ±0.005	-0.12 ±0.02	10 ± 2	-0.011 ±0.033
Pteropus	-0.118 ±0.014	0.055 ±0.008	-0.10 ±0.02	0 ± 2	-0.080 ±0.015
Pteranodon	0.054 ±0.023	0.004 ±0.018	-0.07 ±0.04	5 ± 3	-0.050 ±0.029
Pterodactylus	0.200 ±0.020	-0.050 ±0.009	-0.05 ±0.03	0 ± 4	-0.040 ±0.009
Rhamphorhynchus	-0.062 ±0.013	-0.192 ±0.024	-0.05 ±0.01	15 ± 1	-0.018 ±0.044
Sphere	-0.037 ±0.023	-0.020 ±0.022	-0.03 ±0.01		-0.050 ±0.006
Weathervane	-0.333 ±0.040	-0.347 ±0.020	-0.03 ±0.04	0 ± 2	-0.210 ±0.055

	b			c		
	$dC_m/d\delta$, tail dorsiflexion[1]			$dC_m/d\delta$, sym protraction / wing sweep[2]		
	$\alpha = 0°$	$\alpha = 15°$	$\alpha = 75°$	$\alpha = 0°$	$\alpha = 15°$	$\alpha = 75°$
Anchiornis	0.168 ±0.002	0.191 ±0.006	0.047 ±0.012	0.00±0.020	0.050±0.020	0.070 ±0.004
Archaeopteryx	0.219 ±0.007	0.190 ±0.010	0.065 ±0.024	-0.003 ±0.016	0.060 ±0.018	0.128 ±0.004
Confuciusornis	0.011 ±0.003	0.013 ±0.003	0.018 ±0.005	0.006 ±0.034	0.117 ±0.023	0.229 ±0.002
Jeholornis	0.268 ±0.019	0.223 ±0.001	0.068 ±0.005	0.042 ±0.009	0.072 ±0.006	0.088 ±0.002
Microraptor	0.174 ±0.023	0.125 ±0.002	0.089 ±0.014	0.018 ±0.008	0.037 ±0.003	0.051 ±0.004
Sapeornis	0.054 ±0.001	0.064 ±0.005	0.082 ±0.006	0.022 ±0.038	0.173 ±0.039	0.329 ±0.001
Zhongjianornis	0.023 ±0.004	0.019 ±0.004	0.012 ±0.026	0.025 ±0.027	0.119 ±0.023	0.221 ±0.001
Alectoris	0.011 ±0.004	0.012 ±0.005	0.044 ±0.007	-0.001 ±0.017	0.124 ±0.020	0.119 ±0.002
Buteo	0.018 ±0.003	0.033 ±0.012	0.049 ±0.006	0.043 ±0.034	0.142 ±0.021	0.213 ±0.001
Columba	0.044 ±0.004	0.050 ±0.003	0.009 ±0.002	0.076 ±0.027	0.151 ±0.013	0.168 ±0.002
Larus	0.014 ±0.008	0.016 ±0.003	0.014 ±0.008	-0.007 ±0.030	0.110 ±0.032	0.231 ±0.001
Onychonycteris	0.029 ± 0.004	0.032 ± 0.002	0.004 ± 0.004	0.031 ±0.030	0.152 ±0.031	0.235 ±0.003
Pteropus	0.053 ± 0.009	0.026 ± 0.003	0.026 ± 0.002	-0.005 ±0.041	0.131 ±0.030	0.230 ±0.002
Pteranodon	0.016 ±0.005	0.015 ±0.002	0.016 ±0.003	0.167 ±0.050	0.315 ±0.033	0.480 ±0.001
Pterodactylus	0.015 ±0.002	0.025 ±0.004	0.039 ±0.005	0.005 ±0.026	0.088 ±0.018	0.141 ±0.002
Rhamphorhynchus	0.347 ±0.016	0.245 ±0.032	0.029 ±0.010	0.012 ±0.025	0.045 ±0.020	0.175 ±0.001

[1]movement depicted in Figure 3b
[2]movement depicted in Figure 3a

Table S2. (a) Roll static stability and (b) control effectiveness in roll for asymmetric wing tucking (Figure 3c). Results give mean ± s.d. for $n = 15$; units for stability and control effectiveness are rad^{-1}. Equilibrium point in roll is at $\phi = 0°$.

	a		b	
	roll stability, $dC_r/d\phi$		$dC_r/d\delta$, asymmetric wing tuck[1]	
	$\alpha = 15°$	$\alpha = 75°$	$\alpha = 15°$	$\alpha = 75°$
Anchiornis				
Archaeopteryx	0.009 ± 0.06	-0.200 ± 0.019	0.090 ± 0.005	0.200 ± 0.018
Confuciusornis	-0.020 ± 0.02	-0.200 ± 0.009	0.050 ± 0.002	0.100 ± 0.012
Jeholornis	0.073 ± 0.03	-0.400 ± 0.025	0.080 ± 0.004	0.120 ± 0.024
Microraptor	0.132 ± 0.03	-0.300 ± 0.019	0.050 ± 0.007	0.170 ± 0.021
Sapeornis	0.043 ± 0.04	-0.300 ± 0.026	0.080 ± 0.002	0.130 ± 0.016
Zhongjianornis	0.030 ± 0.02	-0.200 ± 0.012	0.050 ± 0.001	0.140 ± 0.015
Alectoris	0.009 ± 0.06	-0.100 ± 0.016	0.060 ± 0.002	0.080 ± 0.007
Buteo	0.028 ± 0.05	-0.400 ± 0.022	0.170 ± 0.012	0.240 ± 0.055
Columba	-0.030 ± 0.05	-0.300 ± 0.014	0.180 ± 0.007	0.180 ± 0.027
Larus	-0.009 ± 0.02	-0.400 ± 0.028	0.150 ± 0.004	0.200 ± 0.038
Onychonycteris		-1.000 ± 0.044	0.810 ± 0.036	0.880 ± 0.100
Pteropus	-0.027 ± 0.05	-0.700 ± 0.064	0.680 ± 0.020	0.830 ± 0.088
Pteranodon	0.011 ± 0.02	-0.200 ± 0.014	0.070 ± 0.002	0.100 ± 0.010
Pterodactylus	-0.002 ± 0.06	-0.300 ± 0.016	0.100 ± 0.003	0.110 ± 0.027
Rhamphorhynchus	-0.069 ± 0.03	-0.400 ± 0.021	0.160 ± 0.007	0.210 ± 0.030

[1]movement depicted in Figure 3c

Table S3. (a) Yaw static stability, (b) control effectiveness in yaw using tail lateral flexion (Figure 3d), (c) control effectiveness in yaw using wing pronation/supination (Figure 3e), (d) control effectiveness in yaw using lateral head flexion for pterosaurs only. Results give mean ± s.d., $n = 15$ for stability, $n = 5$ for control effectiveness; units for stability and control effectiveness are rad^{-1}. Equilibrium point in yaw is at $\psi = 0°$.

a

	yaw stability, $dC_y/d\psi$ $\alpha = 15°$	yaw stability, $dC_y/d\psi$ $\alpha = 75°$
Anchiornis	-0.097 ± 0.003	0.006 ± 0.006
Archaeopteryx	-0.070 ± 0.004	0.010 ± 0.004
Confuciusornis	-0.026 ± 0.002	0.004 ± 0.002
Jeholornis	-0.091 ± 0.003	0.002 ± 0.001
Microraptor	-0.100 ± 0.016	0.039 ± 0.010
Sapeornis	0.002 ± 0.003	0.005 ± 0.001
Zhongjianornis	0.021 ± 0.003	0.008 ± 0.002
Alectoris	0.022 ± 0.001	0.001 ± 0.002
Buteo	0.027 ± 0.006	-0.002 ± 0.004
Columba	0.048 ± 0.002	0.003 ± 0.002
Larus	0.017 ± 0.004	0.002 ± 0.002
Onychonycteris	0.025 ± 0.008	-0.004 ± 0.007
Pteropus	0.040 ± 0.025	-0.016 ± 0.005
Pteranodon	0.026 ± 0.002	0.002 ± 0.001
Pterodactylus	-0.002 ± 0.001	0.002 ± 0.001
Rhamphorhynchus	-0.052 ± 0.004	-0.034 ± 0.004

	b $dC_y/d\delta$, lateral tail flexion[1]		c $dC_y/d\delta$, wing pro/sup[2]		d $dC_y/d\delta$, lateral head flexion	
	$\alpha = 15°$	$\alpha = 75°$	$\alpha = 15°$	$\alpha = 75°$	$\alpha = 15°$	$\alpha = 75°$
Anchiornis	0.239 ± 0.070	0.069 ± 0.013	0.199 ± 0.020	0.330 ± 0.004		
Archaeopteryx	0.220 ± 0.071	0.066 ± 0.004	0.420 ± 0.015	0.383 ± 0.016		
Confuciusornis	0.002 ± 0.008	-0.004 ± 0.001	0.206 ± 0.025	0.184 ± 0.007		
Jeholornis		-0.027 ± 0.007				
Microraptor	0.520 ± 0.083	-0.076 ± 0.010	0.259 ± 0.013	0.373 ± 0.008		
Sapeornis						
Zhongjianornis	-0.001 ± 0.002	-0.007 ± 0.001	0.296 ± 0.015	0.262 ± 0.015		
Alectoris	0.019 ± 0.012	-0.050 ± 0.001	0.081 ± 0.013	0.093 ± 0.004		
Buteo	-0.007 ± 0.003	-0.029 ± 0.003	0.565 ± 0.060	0.431 ± 0.025		
Columba	0.005 ± 0.002	-0.022 ± 0.001	0.455 ± 0.042	0.204 ± 0.003		
Larus		-0.012 ± 0.002				
Onychonycteris	-0.011 ± 0.005	-0.012 ± 0.003	0.870 ± 0.093	0.627 ± 0.049		
Pteropus						
Pteranodon			0.271 ± 0.013	0.234 ± 0.004	0.120 ± 0.002	-0.003±0.001
Pterodactylus			0.196 ± 0.014	0.139 ± 0.013	0.190 ± 0.003	0.002±0.001
Rhamphorhynchus	0.170 ± 0.008	0.128 ± 0.002	0.279 ± 0.027	0.319 ± 0.024	-0.033±0.009	

[1]movement depicted in Figure 3d [2]movement depicted in Figure 3e

Table S4. Fossil paravians (Xu et al., 2011) sampled for aerodynamic testing and references used during model construction.

	specimen and reference	approx length $\times 10^{-2}$ m
Anchiornis	LPM B00169 (Hu et al., 2009)	42
Archaeopteryx	Berlin (Benton, 2005; Longrich, 2006)	40
Confuciusornis	multiple (Hou et al., 1995; Chiappe et al., 1999)	30
Jeholornis	IVPP V13274, 13553 (Zhou et al., 2002; Zhou and Zhang, 2003b; O'Connor et al., 2013)[1]	65
Microraptor	IVPP V13352 (Xu et al., 2003; Li et al., 2012)	85
Sapeornis	IVPP V13275 (Zhou and Zhang, 2003a; Zheng et al., 2013)	27
Zhongjianornis	IVPP V15900 (Zhou and Li, 2010)	22

[1] feathers only in 13553

Table S5. Geometry data for physical models of eight fossil paravians, four extant birds, two bats, three pterosaurs, and two shapes for checking calibration. Aspect ratio (AR) calculated as s^2/S.

	area, S $\times 10^{-4}$ m^2	SVL $\times 10^{-2}$ m	TL $\times 10^{-2}$ m	span, s $\times 10^{-2}$ m	AR
Anchiornis	87.11	7.1	18.0	19.6	4.4
Archaeopteryx	94.57	8.0	10.5	17.7	3.4
Confuciusornis	50.53	6.8	9.2	19.9	7.8
Jeholornis	77.03	7.7	19.0	22.7	6.8
Microraptor	114.6	9.3	22.5	19.2	3.2
Sapeornis	54.44	6.6	7.6	20.7	7.8
Zhongjianornis	61.87	8.3	10.1	21.3	7.4
Alectoris	57.89	7.1	9.8	15.0	4.0
Buteo	98.55	8.3	9.9	23.8	5.8
Columba	80.71	7.3	9.8	19.3	4.6
Larus	72.62	7.9	10.5	24.0	8.0
Onychonycteris	194.7	9.6	13.4	29.5	4.4
Pteropus	201.2	8.4	12.4	35.1	6.2
Pteranodon	42.13	6.4	6.5	22.1	11.6
Pterodactylus	51.15	8.4	8.9	19.0	7.0
Rhamphorhynchus	78.56	8.0	18.4	29.7	11.2
Sphere	11.34		3.8	3.8	1.3
Weathervane	39.30		24.0	5.0	0.6
