# Supplementary figures and images for: Shifts in stability and control effectiveness during evolution of Paraves support aerial maneuvering hypotheses for flight origins
Source: PeerJ. 2014 Oct 16;2:e632. doi: 10.7717/peerj.632 (PMC4203027; doi:10.7717/peerj.632)

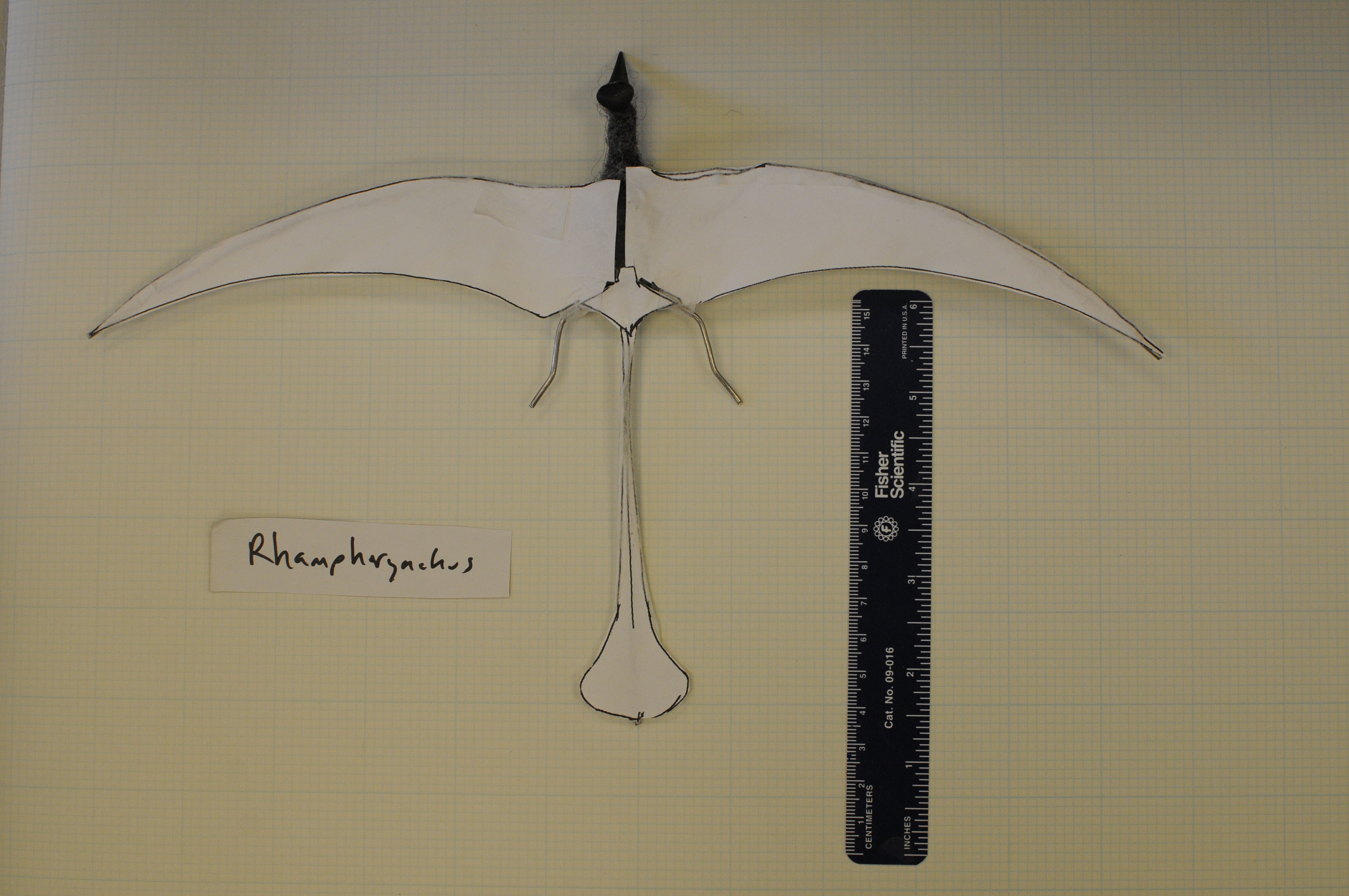

Supplement: Supplemental Information 4 [file peerj-02-632-s004.gz › additional-photos/_DSC5205.JPG]

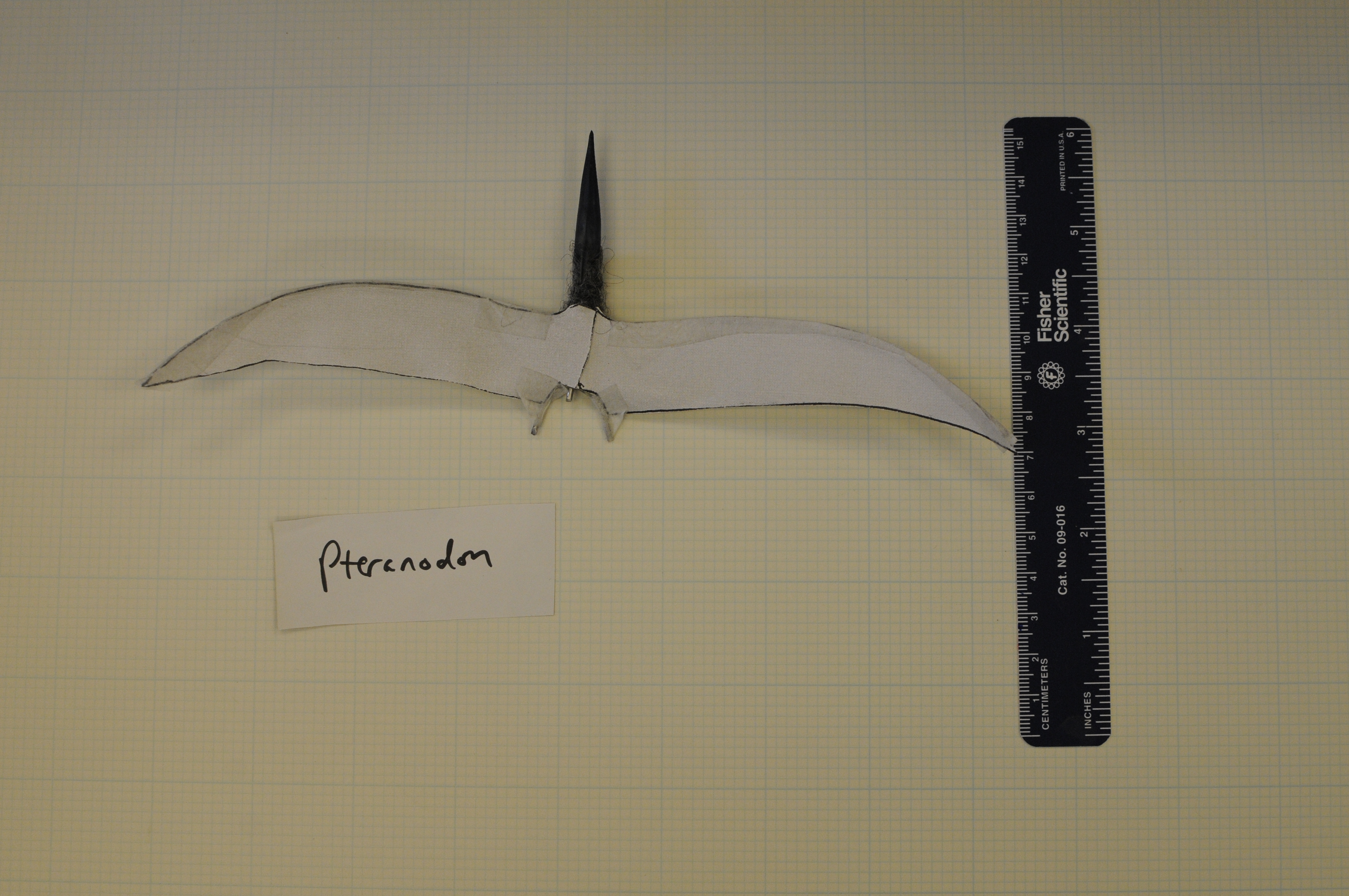

Supplement: Supplemental Information 4 [file peerj-02-632-s004.gz › additional-photos/_DSC5201.JPG]

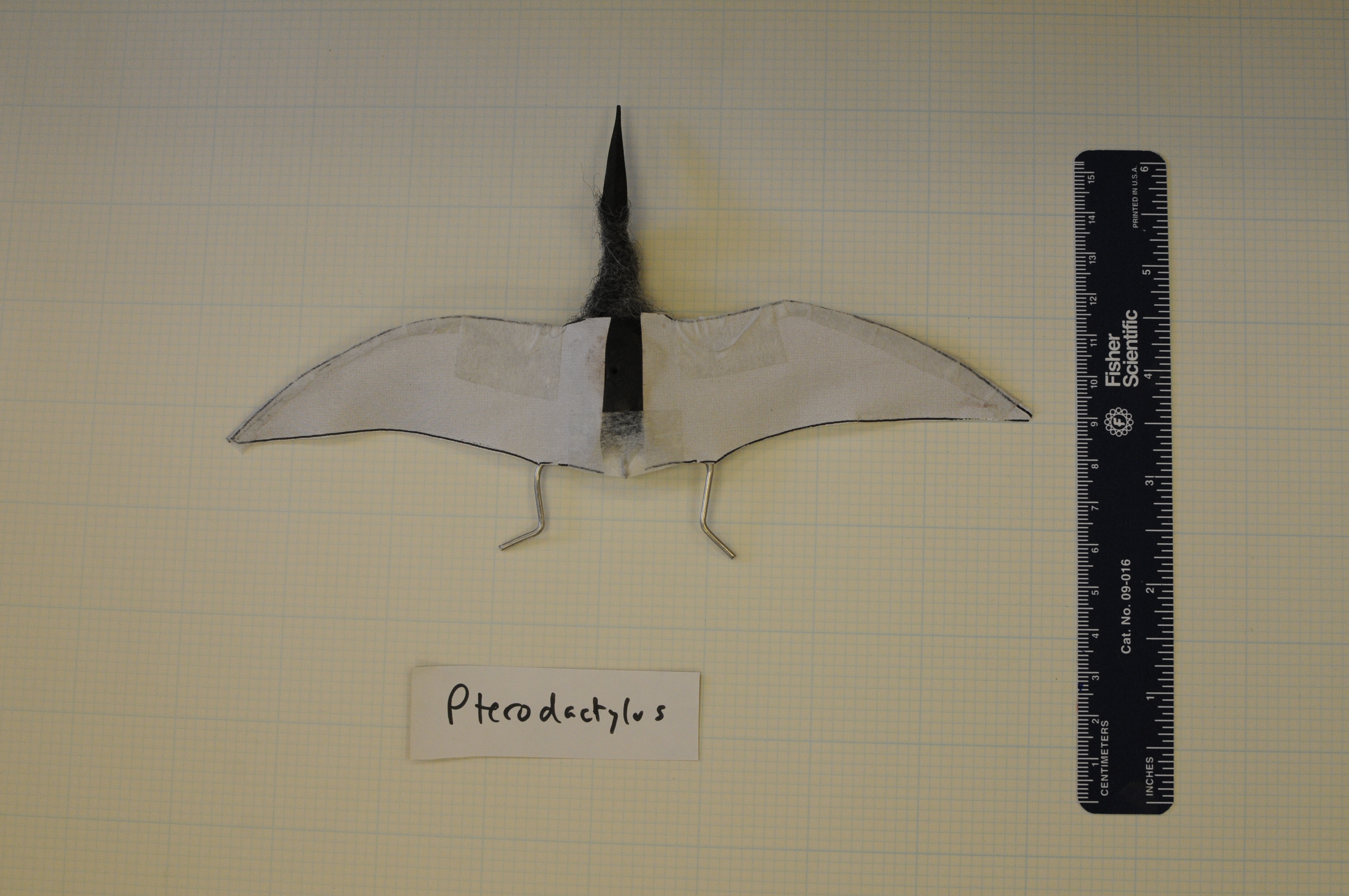

Supplement: Supplemental Information 4 [file peerj-02-632-s004.gz › additional-photos/_DSC5203.JPG]

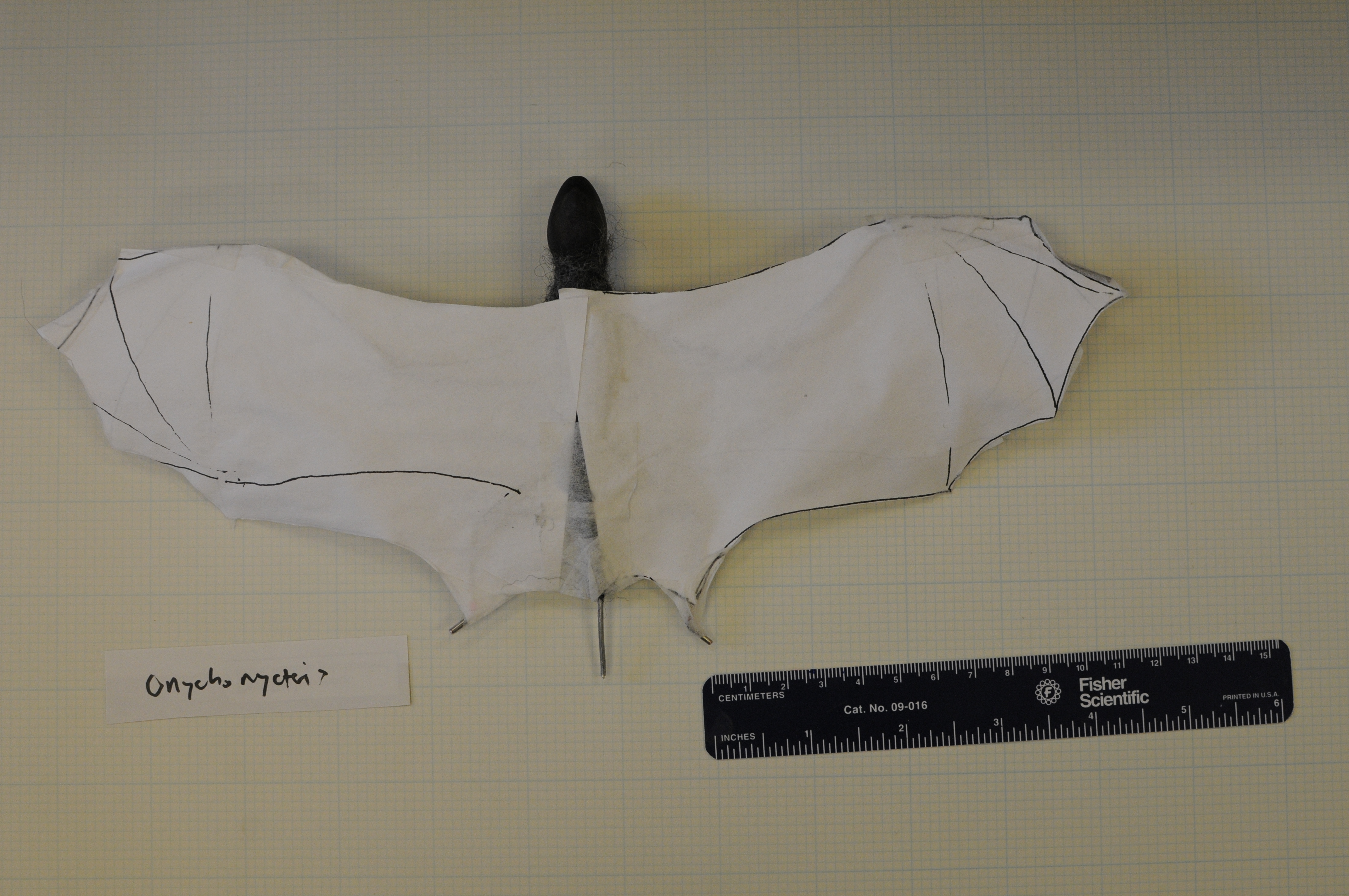

Supplement: Supplemental Information 5 [file peerj-02-632-s005.gz › additional-photos/_DSC5209.JPG]

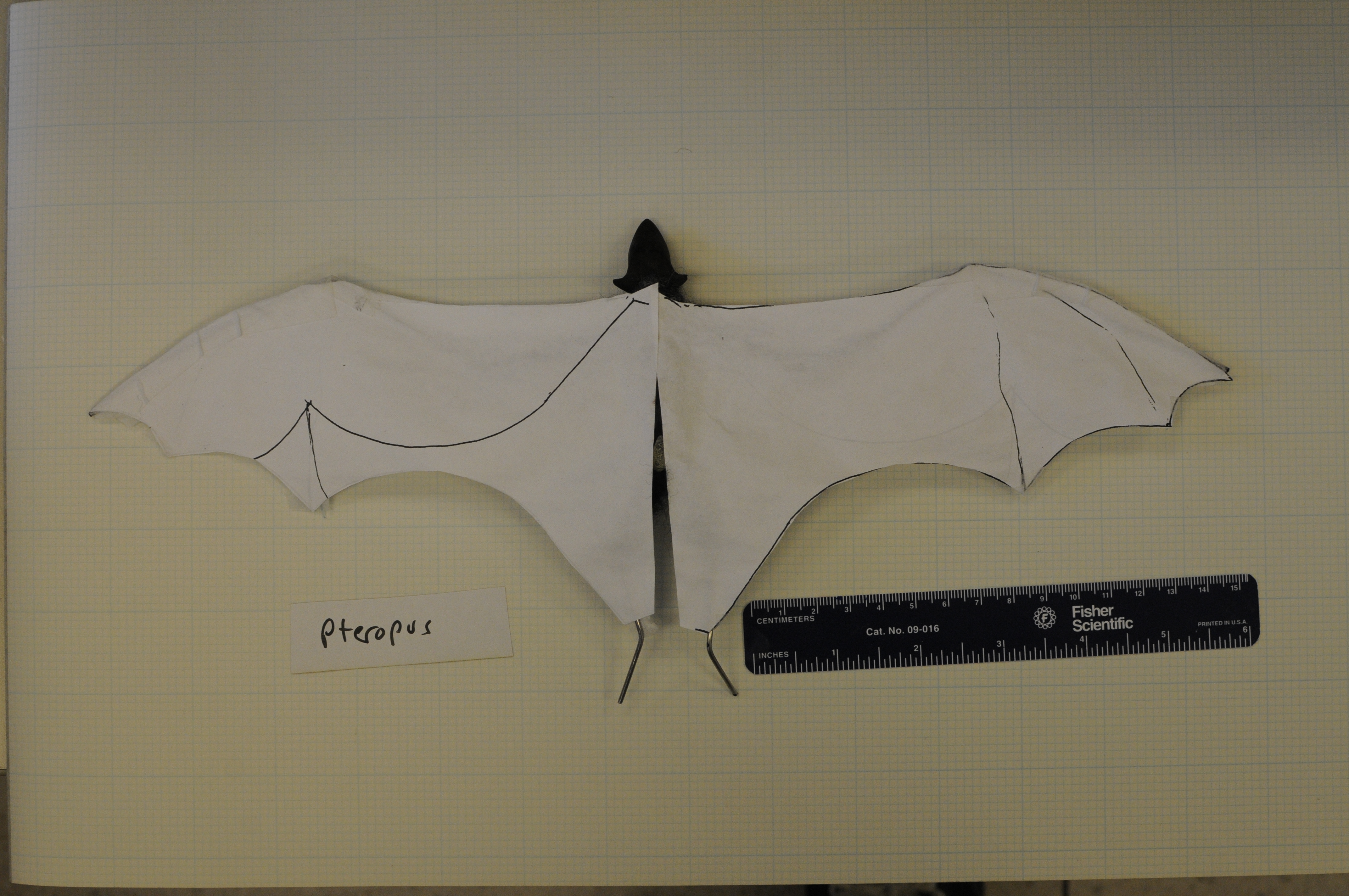

Supplement: Supplemental Information 5 [file peerj-02-632-s005.gz › additional-photos/_DSC5207.JPG]
